# Machine Learning Prediction of Protein Adsorption on Drug-delivering Nanoparticles: A Literature Survey and Need for Future Development

**DOI:** 10.1007/s11095-025-03981-6

**Published:** 2025-11-26

**Authors:** Koushiki Basu, Venkata S. Chelagamsetty, Veronica A. Ruiz-Avila, Tonglei Li

**Affiliations:** Department of Industrial and Molecular Pharmaceutics, Purdue University, 575 Stadium Mall Dr., West Lafayette, IN 47907 USA

**Keywords:** deep learning, drug delivery, machine learning, nanoparticles, protein adsorption, protein corona, random forest

## Abstract

Nanoparticles (NPs), due to their small size and large surface area, have advanced their use as drug carriers for delivering various therapeutic molecules. When entering biological environments, nanoparticles typically adsorb proteins, forming a surface layer known as a protein corona that significantly affects the biological and therapeutic functions of a delivery system. Understanding and predicting protein adsorption is essential for optimizing nanoparticle design in drug delivery, diagnostics, and therapy. Machine learning and deep learning (ML/DL) offer promising methods for designing nanoparticles with specific properties, particularly given recent advancements in computation and nanoparticle analysis. This review explores ML/DL studies of nanoparticle-protein interactions and emphasizes the popularity of Random Forest (RF) and Deep Learning (DL) models in predicting protein corona compositions. RF models are highly valued for managing high-dimensional data and offering interpretability, which helps identify key NP features influencing protein adsorption. Conversely, DL excels at modeling non-linear relationships and detecting subtle interaction patterns. While most current research focuses on protein coronas, future models may also include other biocorona components. This is particularly relevant for soft materials, such as lipid nanoparticles (LNPs), which are now approved for delivering mRNA and peptide-based vaccines. Our findings underscore the need for advanced modeling techniques and high-quality, diverse experimental data to drive innovations in nanomedicine. Combining RF and DL approaches leverages their complementary strengths to overcome the challenge of limited experimental data and further improve NP designs for biomedical use.

**Graphical Abstract:**

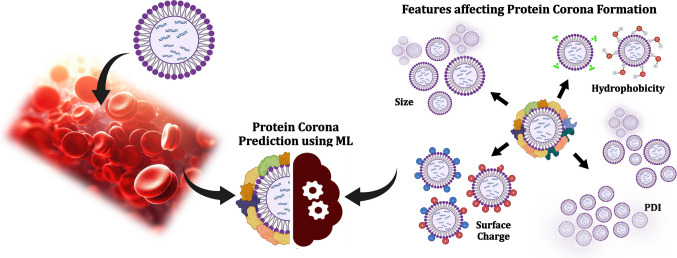

## Introduction

Nanoparticles are minute particles characterized by size-dependent properties, with at least one dimension ranging from 1 to 100 nm [1]. These particles possess unique physical, chemical, and biological properties, and due to their small size, large surface area relative to their volume, and the ability to be functionalized with various biomolecules, they have led to the development of novel innovations spanning a broad range of fields [2–5]. In particular, these attributes make them especially valuable in biomedicine, where nanoparticles play a central role in targeted drug delivery and theranostics, facilitating improved disease detection and treatment, particularly in oncology [6–11]. Several classes of nanocarriers, including metallic NPs, liposomes, polymeric NPs, and dendrimers, have been investigated for clinical translation. Metallic nanoparticles (gold, silver, and iron oxide NPs), are particularly well represented in nanomedicine databases and are valued for their distinctive features, including localized surface plasmon resonance and superparamagnetism, which underpin applications in imaging, biosensing, photothermal cancer therapy, and antimicrobial treatments [12–14]. Recently, lipid nanoparticles (LNPs) have emerged as a leading class of clinically approved nanocarriers, particularly following their successful use in delivering mRNA vaccines against COVID-19 [15–17]. Beyond vaccines, LNPs are increasingly studied for the delivery of nucleic acids, proteins, and peptides, where their tunability and biocompatibility offer clear advantages [18, 19]. A central challenge for both metallic and lipid-based nanocarriers is their interaction with endogenous proteins, which governs biodistribution, stability, and therapeutic outcomes. Understanding and predicting these protein–nanoparticle interactions is therefore crucial for the rational design of nanomedicine.

When NPs enter biological environments such as blood, plasma, or cytoplasm, they rapidly adsorb a dynamic layer of endogenous proteins, known as the protein corona [20], that fundamentally alters their physicochemical and biological identity, thereby dictating their *in vivo* fate [20, 21]. This corona consists of a tightly bound ‘hard’ layer and a more dynamic ‘soft’ layer, whose composition and kinetics depend on nanoparticle size, shape, charge, surface chemistry, and the surrounding protein milieu [22–31]. Critically, the protein corona governs nanoparticle biodistribution by mediating recognition by biological systems. For instance, in LNPs, the adsorption of apolipoproteins (ApoE) enables binding to low-density lipoprotein receptors on hepatocytes, facilitating liver-specific uptake, which is essential for the success of mRNA therapeutics [32]. Beyond targeting, the corona modulates immune interactions, as specific protein components can cloak particles from phagocytic clearance by the mononuclear phagocyte system, thereby extending circulation times. In contrast, others promote opsonization and accelerate clearance [33–35]. Additionally, the physicochemical properties of nanoparticles influence corona formation and biodistribution; for example, softer nanoparticles tend to adsorb a more diverse protein corona, which correlates with reduced macrophage uptake compared to stiffer particles. The corona is also highly dynamic and context-dependent, evolving as particles traverse different biological compartments, with proteins continuously exchanging between the hard and soft layers in response to local anatomical and biochemical environments. Together, these multifaceted interactions highlight the critical importance of understanding protein corona composition and dynamics for designing nanocarriers with predictable biodistribution, immune evasion, and therapeutic efficacy [36, 37].

Protein adsorption onto NP surfaces is driven by the overall decrease in system free energy, orchestrated by several molecular-level interactions. Electrostatic forces between charged amino acid residues and nanoparticle surface moieties initiate adsorption, their strength modulated by the nanoparticle’s charge and the protein’s isoelectric point, as well as ionic conditions in solution [38–40]. Hydrophobic interactions typically dominate by driving proteins to expose nonpolar residues to reduce interfacial tension with aqueous environments, often inducing conformational changes that stabilize adsorbed proteins [41]. Additionally, van der Waals forces collectively contribute to adsorption stability at close distances, while hydrogen bonding between protein functional groups and particle surfaces or hydration layers further refine protein orientation and binding affinity [42]. Charge-transfer interactions may also influence binding energetics through electron donor–acceptor mechanisms. Nanoparticle curvature and surface roughness affect spatial protein packing, modulating adsorption kinetics and corona composition. The relative contributions of these forces depend strongly on the nanoparticle’s surface chemistry and the local environment, such as pH, temperature, and ionic strength [43]. Understanding these synergistic driving forces at the nano-bio interface is crucial for engineering nanoparticle surfaces that control protein corona formation, thereby enhancing the performance of nanomedicine.

Characterizing the protein corona is essential for understanding and optimizing NP behavior. To assess physical changes, techniques such as Dynamic Light Scattering (DLS) and zeta potential detect size and charge variations, while Transmission Electron Microscopy (TEM) and Atomic Force Microscopy (AFM) provide visualization of morphology and corona thickness. Complementing these, mass spectrometry and related protein analytic methods yield detailed information on corona composition, structure, and abundance, which is particularly important for evaluating antigen presentation and immune activation in vaccine delivery. Together, the growing body of experimental data from these techniques, combined with advances in machine learning (ML), provides a powerful opportunity to predict NP–protein interactions and guide the rational design of NP-based drug delivery systems [44–46].

Machine learning (ML), a pivotal domain of Artificial Intelligence (AI), leverages statistical models to identify patterns and make predictions from experimental data with minimal human intervention [47–49]. ML approaches are broadly categorized as supervised, unsupervised, semi-supervised, and reinforcement learning, each suited for specific problem types [50–52]. Supervised learning, the most common, uses labeled datasets for regression or classification tasks, with algorithms ranging from decision trees and SVMs to deep learning (DL) neural networks. DL, employing multi-layered architectures such as CNNs for images or RNNs or LSTMs for sequential data, excels at handling complex, high-dimensional datasets [53, 54]. Unsupervised learning uncovers hidden structures in unlabeled data through clustering or dimensionality reduction. In contrast, semi-supervised learning combines the strengths of both approaches by blending small labeled datasets with large unlabeled datasets to improve predictive accuracy [55–57]. Lastly, reinforcement learning optimizes decisions by iteratively interacting with environments and receiving feedback through rewards or penalties [58, 59]. The ML workflow involves data curation, molecular representation, model selection, training, validation, and interpretation. High-quality, diverse datasets are critical, with molecular features (fingerprints, graphs, descriptors) encoding chemical information for model input. Statistical and deep learning models are then trained to predict biological outcomes, validated for generalization, and interpreted to uncover structural drivers of activity [60–62]. ML has been widely applied in drug discovery, toxicology, materials science, and increasingly in nanomedicine to predict protein corona formation on nanocarriers, such as LNPs, facilitating the optimization of biodistribution and immunogenicity [63–69]. Despite their significant utility, ML/DL models face several critical challenges, including data quality and bias, limited labeled datasets, the complexity of biological systems, the representation of molecular and nanomaterial features, the interpretability of deep models, and the risk of overfitting [70]. High-quality data in this context means accurate and reproducible measurements with well-defined experimental conditions and metadata, consistent feature annotation, and minimal missing values. Large, diverse datasets are equally critical for capturing the chemical and biological variability of nanomaterials and avoiding bias toward overrepresented nanoparticle types or experimental settings. Addressing these challenges requires rigorous data preprocessing, diverse and high-fidelity datasets, and interdisciplinary strategies that combine experimental and computational validation. Overcoming these limitations is crucial for developing robust, predictive models that can guide rational design in drug delivery and nanomedicine.

**Fig. 1:**
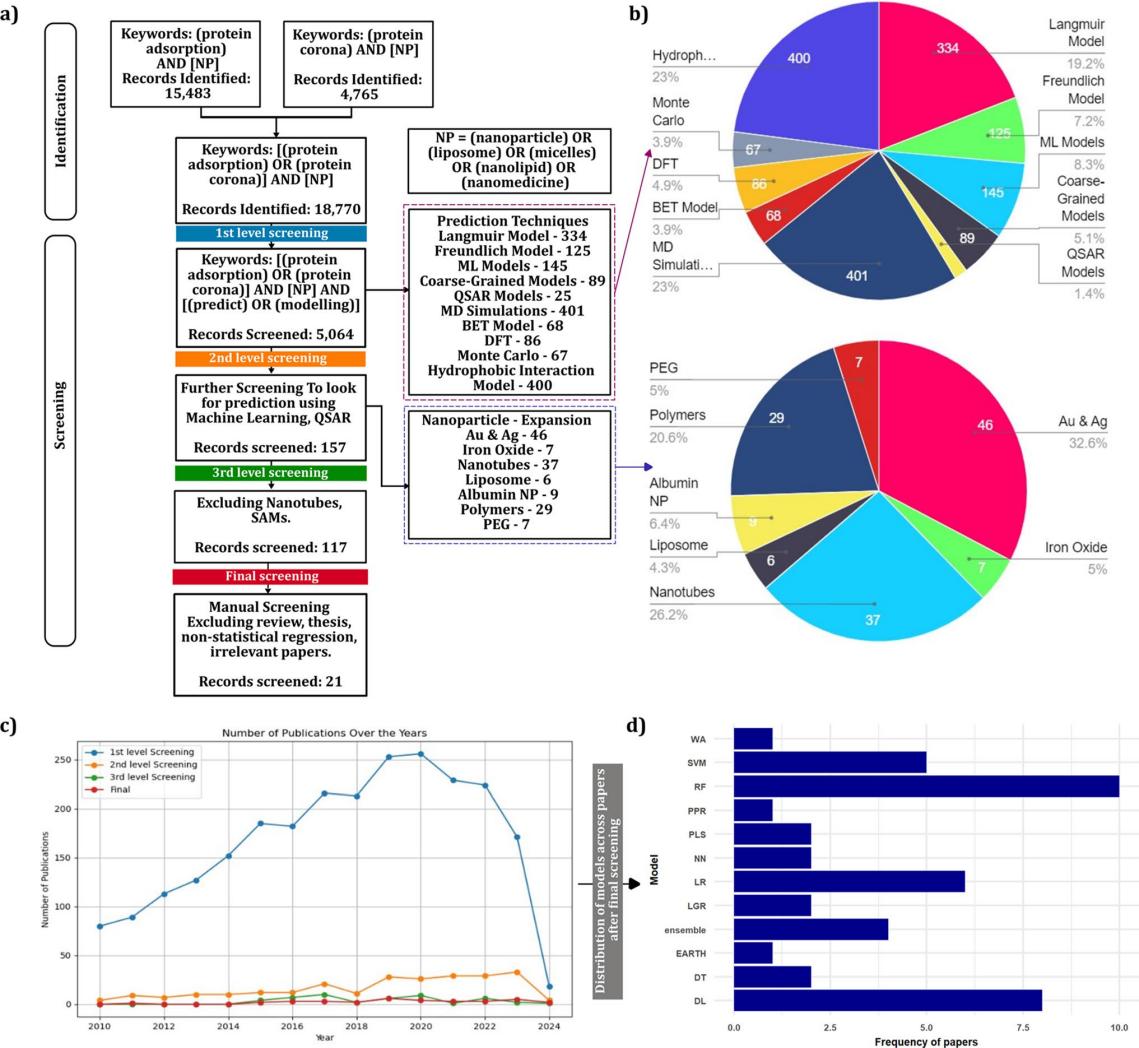
Our literature search steps and findings: (**a**) flow diagram of the search strategy, (**b**) number of papers screened following different prediction techniques and different nanoparticles, (**c**) number of publications over the years at each level of screening, and (**d**) distributions of different ML models across papers after final screening.

This work aims to comprehend how ML/DL approaches have been applied to understand protein adsorption and corona formation in nanoparticle-based drug delivery. As outlined in Fig. 1a, a systematic review was conducted using the Web of Science core database (Spring 2024) with keywords including “protein adsorption,” “protein corona,” “nanoparticle,” and “modeling.” After applying inclusion and exclusion criteria, 21 eligible research articles (2011–2024) were identified [19, 22, 71–89]. Figure 1b visualizes the distribution of papers focusing on specific predictive approaches and nanoparticle types, giving insight into the diversity across the reviewed literature. Most of them focused on metallic nanoparticles and serum protein interactions under varying conditions, while only a few addressed lipid nanoparticles (LNPs), despite their increasing clinical relevance for vaccines, peptide therapies, and protein-based biologics. The distribution of the publications over the years is highlighted in Fig. 1c, which illustrates the narrowing down of papers as the review progressed from the initial broad search to the final set of articles. The final 21 studies were comprehensively analyzed, with model usage summarized in Fig. 1d and detailed information on study aims, nanoparticle and protein features, measurement techniques, ML models, validation methods, and performance outcomes compiled in Table I. It compiles the ML architectures employed, such as Linear QSAR, Random Forest (RF), Support Vector Machines (SVM), and Artificial Neural Networks (ANN), alongside validation metrics like R^2^, RMSE, precision, and recall. As depicted in Fig. 1, Random Forest (RF) and Deep Learning (DL) were the most frequently used models. This predominance arises because RF is robust to small, heterogeneous datasets and provides interpretable insights into feature importance, while DL excels at capturing complex, nonlinear relationships in high-dimensional and multimodal nanoparticle-protein interaction data. Their complementary strengths make them particularly suitable for modeling protein corona formation, guiding nanoparticle design, and predicting biological outcomes in drug delivery applications. This review highlights experimental and computational gaps that must be addressed to develop reliable predictive models, with special focus on the underexplored lipid nanoparticles used in mRNA vaccines and other biologics. The insights aim to guide researchers in creating high-quality datasets, selecting suitable machine learning methods, and developing interpretable models to more accurately predict protein corona formation and inform the design of nanocarriers.

**Table 1:** Summary Metrics of 21 Machine Learning Studies

Source	Aim of Study	Type of NPs	NP features	Type of protein	Protein features	ML model	Performance measure	Value Range	Model validation
Liu et al. (2015) [71]	NP-cell association = f(protein corona fingerprints, NP physicochemical properties)	84 gold NPs of 15, 30, or 60 nm cores with cationic or anionic surface ligands	As synthesized: Hydrodynamic size, zeta potential, Localized Surface Plasmon Resonance (LSPR) index, LSPR peak position, TEM core size, surface area of NP With serum: Hydrodynamic size, zeta potential, LSPR index, total adsorbed protein, total NP surface area, protein density on NP surface	6 Serum proteins	Not mentioned	Linear QSAR	R^2^	[0.83,0.89]	Leave-one-out validation (Each sample is left out once for testing, rest used for training) and fourfold cross-validation (Dataset split into 4 folds and each fold is used once for testing)
						ε-SVR QSAR	R^2^	[0.85,0.97]	
Bigdeli et al. (2016) [22]	cellular interactions of liposomes = f(protein corona fingerprints, liposome physicochemical properties)	17 liposomes	-HP: size, molecular weight, surface area per liposome, mol, zeta potential + HP: size, zeta potential, protein density	HP protein	Not mentioned	MLR	R^2^	[0.12–0.94]	Monte Carlo cross-validation (Randomly selects training/testing splits multiple times)
							RMSE	[0.04–3.42]	
						ANN	R^2^	[0.01–0.99]	
							RMSE	[0.04–2.99]	
Papa et al. (2016) [72]	NP bioactivity = f(protein corona composition)	105 surface-modified Au-NPs	- Molecular properties: zav_serum, which is the hydrodynamic diameter measured after exposure to serum -Spectral counts of proteins [A1AT (Alpha 1 antitrypsin), CO4B (Complement C4B), KNG1 (Kininogen-1), VNTC (Vitronectin), and GFAP (Glial Fibrillary Acidic Protein)]. A1AT, VNTC, and COB4 function as promoters of the cellular response, while KNG1 and GFAP act as inhibitors of the modeled response	785 serum protein	Not mentioned	k-NN	R^2^	[0.73,0.88]	Leave-one-out cross-validation
							RMSE	[0.81,1.17]	
						GregNN	R^2^	[0.74,0.93]	
							RMSE	[0.63,1.18]	
						RBFNN	R^2^	[0.74,0.87]	
							RMSE	[0.82,1.10]	
						CPANN	R^2^	[0.82,0.92]	
							RMSE	[0.66,1.25]	
						SVM-radial	R^2^	[0.76,0.94]	
							RMSE	[0.59,1.09]	
						SVM-linear	R^2^	[0.78,0.87]	
							RMSE	[0.82,1.04]	
						PLS	R^2^	[0.76,0.87]	
							RMSE	[0.81,1.07]	
						MLR	R^2^	[0.76,0.87]	
							RMSE	[0.81,1.07]	
						PPR	R^2^	[0.79,0.91]	
							RMSE	[0.69,1.01]	
						EARTH	R^2^	[0.80,0.90]	
							RMSE	[0.73,1.10]	
						RF-6	R^2^	[0.80,0.95]	
							RMSE	[0.62,1.29]	
						RF-150	R^2^	[0.80,0.95]	
							RMSE	[0.63,1.43]	
Fojnica et al. (2017) [73]	protein concentration adsorbed on Au-NPs = f(free protein concentration)	210 Au NPs	Not mentioned	sIgG protein	Free protein concentration	ANN	Sensitivity	92.50%	validated on 90 samples, 80 of them were known protein concentrations, and 10 of them were used as blank to check whether the system can distinguish between control samples and protein concentration
							Specificity	100.00%	
Helma et al. (2017) [74]	NP toxicology = f(structural descriptors, physicochemical NP properties, biological NP properties)	121 nanoparticles in the Protein Corona dataset (gold and silver particles) from eNanoMapper (integrated platform designed for the characterization and predictive modeling of engineered nanomaterials)	- Structural descriptors: Union of MOLPRINT (molecular fingerprinting technique that encodes chemical substructures) 2D fingerprints for core and coating compounds - Physico-chemical nanoparticle properties: Measured nanoparticle properties from the eNanoMapper database - Biological nanoparticle properties: Protein interaction data from the eNanoMapper database	Human serum	Not mentioned	WA	R^2^	[0.19–0.70]	5 repeated tenfold cross-validation (tenfold repeated 5 times with different splits)
							RMSE	[1.44–2.07]	
						PLS	R^2^	[0.27–0.67]	
							RMSE	[1.55–2.16]	
						RF	R^2^	[0.45–0.69]	
							RMSE	[1.51–2.10]	
Findlay et al. (2018) [75]	protein corona populations = f(biophysicochemical characteristics of proteins, ENM properties, solution conditions)	Silver ENMs	Size and surface coating	962 unique yeast proteins	pI, protein weight, protein abundance, %positive amino acids, %negative amino acids, %hydrophilic amino acids, %aromatic amino acids, %cysteine, InterPro number, enzyme commission number	RF	Precision	[0.76,0.80]	fivefold cross-validation
							Recall	[0.86, 0.91]	
							Accuracy	[0.54,0.95]	
							F1-score	[0.81,0.85]	
							AUROC	[0.83,0.86]	
Magro et al. (2018) [76]	Normalized peak intensity of peptide detected by MS = f(surface characteristics of SAMNs)	SAMNs	High or low SCC	Peptide in bovine milk	Not mentioned	LGR	Accuracy	90.00%	Leave-one-out cross-validation
Curtis et al. (2019) [77]	NP properties and aspects of the brain microenvironment = f (large trajectory datasets)	PS-COOH and PS-PEG	< MSD > profiles, raw D_eff_ distribution, 17 other distinct features	Horse Serum Proteins	Not mentioned	ANN	Recall	[0.07–1.00]	Leave-one-out cross-validation
Du et al. (2019) [78]	Protein corona formation = f(environmental sulfidation which is an aging process)	Ag and ZnO NPs	Not mentioned	> 250 proteins, majorly HSA	Molecular weight, isoelectric point (pI), GRAVY value, content of H-bonding relevant amino acids (serine, threonine, asparagine, glutamine), content of cysteine	RF	Accuracy	~ 71.00%	fivefold cross-validation
							F1-score	~ 68.00%	
							Error	~ [36.00%, 43.00%]	
Movahedi et al., 2019 [79]	Concentration of specific proteins adsorbed on NP surface = f(physicochemical properties of NPs)	CeO2, Si-CeO2, BaSO4, and ZnO	SSA, primary particle size, hydrodynamic diameter, polydispersity index, zeta potential, density	3 (C3), complement factor B (Cfb), Transferrin (Tf), Albumin, α1 antitrypsin (A1AT), Tubuline Beta 2 A Class lia (Tubb2A), Surfactant protein-D (SP-D), Surfactant protein-A (SP-A)	Not mentioned	MLR	MSE	[0.09,1.81]	all—one to six—combinations of features evaluated on 9 proteins
To et al. (2019) [80]	Developmental toxicity profile in an embryonic zebrafish model = f(ENM physicochemical properties)	15 ENMs	Aspect Ratio, Circularity, Conductance, Median Feret Diameter, Effective Density, Hydrodynamic Diameter, Polydispersity Index, Average Pore Size, Total Pore Volume, Roundness, Surface Area, Zeta Potential	Not mentioned	Not mentioned	RFDT	R^2^	[−0.05,0.69]	set of three silver nanoparticles (10, 20, 30nm) was used to assess the model performance
							MSE	[1.10,6.48]	
Ban et al. (2020) [81]	Functional protein composition in the protein corona on NP = f(physicochemical properties of NPs, environmental characteristics)	Various NPs (carbonaceous, metallic, nonmetallic, liposomal, anionic, cationic, neutral, etc.)	8 qualitative factors (NP type, shape, NP without modification, surface modification, modification type, dispersion medium, incubation plasma source, incubation culture) 13 quantitative factors (size by TEM, size by DLS, dispersion medium pH, zeta potential, polydispersity index, incubation plasma concentration, incubation NP concentration, incubation time, incubation temperature, centrifugation speed, centrifugation time, centrifugation temperature, centrifugation repetitions)	Apolipoproteins, complement proteins, coagulation proteins, immune proteins, clusterin	theoretical isoelectric point (pI), length, molecular weight, aliphatic index, cysteine content, (GRAVY) score, and protein function	RF	R^2^	[0.61,0.88]	tenfold cross-validation
							RMSE	[1.30%, 10.40%]	
Duan et al. (2020) [82]	protein corona compositions = f(fluorescence change)	15 metallic, 3 cellulose-based, & 4 2D ENMs (Graphene, hexagonal boron nitride (hBN), & molybdenum disulfide (MoS2))	Size, Charge, Fluorescence Change of different proteins with ENMs measured at 37, 60, 80℃	Human serum proteins	Isoelectric point (pI), molecular weight (Mw), GRAVY, percentage of negative/positive/aromatic amino acids	RF Classification	Precision	[0.66,0.88]	Jackknife cross-validation technique (Leave-one-out like approach, and often applied to feature combinations)
							Recall	[0.56,0.92]	
							F1 score	[0.61,0.88]	
						RF Regression	EVS	[0.03,0.93]	
							R^2^	[0.02,0.95]	
							MAE	[0.26,0.93]	
							MSE	[0.43,2.08]	
Dzisoo et al. (2020) [83]	Hydrophobic interactions of monoclonal antibodies = f(amino acid sequence-based descriptors)	Not mentioned	Not mentioned	131 antibodies were used to develop SSH	Amino acid sequences – 8000 tripeptides (sequence order, total amino acid composition)	SVM	Recall/Sensitivity	[94.595%, 97.297%]	Leave-one-out cross-validation
							Specificity	[81.30%, 87.10%]	
							Accuracy	[89.86%, 92.65%]	
							BAC	[0.89,0.91]	
							AUC	[0.95,0.97]	
							MCC	[0.80,0.86]	
Yan et al. (2020) [84]	Physicochemical properties and biological activities of NPs = f(features derived from NP images)	147 unique NPs, including 123 gold NPs, 12 platinum NPs, and 12 palladium NPs	18 (360°/20°) images were generated for each nanoparticle by taking a screenshot after every 20° rotation around the y-axis. As a result, 2646 images were generated for 147 nanoparticles	Serum Proteins	Not mentioned	CNN	R^2^	[0.68,0.95]	fivefold cross-validation
							RMSE	[0.77,11.29]	
Yu et al. (2021) [85]	Cytotoxicity level of engineered NPs = f(NP properties, experimental conditions, biological conditions)	Cadmium-containing quantum dots (QDs) and Me_x_O_y_ NPs	QD diameter, surface ligand, exposure time, surface modification, cell anatomical type, shell, assay type, surface charge, core, QD source, cell source species, cell origin, delivery type	Not mentioned	Not mentioned	LightGBM	R^2^	[0.72,0.88]	fivefold cross-validation
							RMSE	[0.66,7.25]	
Ouassil et al. (2022) [86]	Likelihood of protein binding to SWCNT = f(protein sequence features)	SWCNT	Not mentioned	Proteins in human blood plasma or cerebro-spinal fluid	Protein features calculated by BioPython and NetSurfP 2.0 (high content of solvent-exposed glycines, nonsecondary structure-associated amino acids, GRAVY score)	LGR	Accuracy	~ 0.64	Stratified shuffle split validation (100 repeats) (Randomly split data multiple times while maintaining class distribution)
							AUC	~ 0.73	
							Precision	~ 0.53	
							Recall	~ 0.56	
						RF (100 or 1000 trees)	Accuracy	~ [0.74,0.76]	
							AUC	~ [0.72,0.73]	
							Precision	~ [0.67,0.68]	
							Recall	~ [0.57,0.58]	
						Bagging (DT, SVM, LGR)	Accuracy	~ [0.67,0.72]	
							AUC	~ [0.70,0.72]	
							Precision	~ [0.51,0.59]	
							Recall	~ [0.52,0.57]	
						GB (DT)	Accuracy	~ 0.72	
							AUC	~ 0.70	
							Precision	~ 0.60	
							Recall	~ 0.56	
						AdaBoost (DT and with 1000 estimators, SVM, LGR)	Accuracy	~ [0.65,0.71]	
							AUC	~ [0.77,0.79]	
							Precision	~ [0.50,0.58]	
							Recall	~ [0.48,0.59]	
						XGBoost (DT and with 100 parallel trees)	Accuracy	~ 0.72	
							AUC	~ 0.71	
							Precision	~ 0.60	
							Recall	~ 0.59	
Chou et al. (2023) [87]	NP delivery efficiency = f(physicochemical characteristics of NP, kinetic parameters)	Collected from “Nano-Tumor Database” (378 tumor datasets from 200 studies after IV administration of different types of NP in tumor-bearing mice)	Types of NPs, core materials of NPs, shape of NPs, hydrodynamic diameter, zeta potential, surface charge	Not mentioned	Not mentioned	LR	R^2^	[−0.08,0.06]	fivefold cross-validation
							RMSE	[2.05,35.28]	
						SVR	R^2^	[−0.18,0.00]	
							RMSE	[2.03,36.80]	
						RF	R^2^	[0.03,0.43]	
							RMSE	[1.75,31.90]	
						XGBoost	R^2^	[0.00,0.36]	
							RMSE	[1.84,32.70]	
						LightGBM	R^2^	[0.03,0.31]	
							RMSE	[1.92,31.70]	
						DNN	R^2^	[0.22,0.91]	
							RMSE	[0.71,32.15]	
de Souza Gama et al. (2023) [88]	Mass front evolution and elution profiles in fixed bed columns = f(thermodynamic-based adsorption isotherms)	SBA-15 (silica-based)	Not mentioned	lysozyme	pH, ionic strength, Lifshitz parameter	DNN	R^2^	[0.94,0.99]	Not mentioned
							MSE	[0.61%, 7.72%]	
Martin et al. (2023) [89]	Cytotoxicity of NPs = f (SiO_2_-NP physicochemical properties, experimental settings, cell type)	Silica (SiO_2_-NP)	Concentration, SiO2-NP medium serum, cell morphology, cell organ, primary size, cell id, exposure time, surface modification, hydrodynamic size water, cell source, assay viability, surface area, viability indicator	Not mentioned	Not mentioned	LDA	Accuracy	[0.65,0.75]	Split-sample internal validation (Simple partition into training and testing sets (e.g., 80:20))
							AUC-ROC	[0.70,0.83]	
							Recall	[0.49,0.64]	
							Precision	[0.38,0.69]	
							nCV_10-fold_	[0.74,0.75]	
						LR	Accuracy	[0.64,0.83]	
							AUC-ROC	[0.64.0.90]	
							Recall	[0.45,0.73]	
							Precision	[0.36,0.78]	
							nCV_10-fold_	[0.74,0.82]	
						Ridge	Accuracy	[0.65,0.75]	
							AUC-ROC	[0.70,0.82]	
							Recall	[0.47,0.64]	
							Precision	[0.38,0.70]	
							nCV_10-fold_	[0.74,0.75]	
						DNN	Accuracy	[0.66,0.76]	
							AUC-ROC	[0.68,0.84]	
							Recall	[0.52,0.67]	
							Precision	[0.36,0.67]	
							nCV_10-fold_	[0.74,0.75]	
						k-NN	Accuracy	[0.74,0.85]	
							AUC-ROC	[0.72,0.83]	
							Recall	[0.67,0.75]	
							Precision	[0.48,0.82]	
							nCV_10-fold_	[0.85,0.86]	
						SVM	Accuracy	[0.76,0.85]	
							AUC-ROC	[0.46,0.89]	
							Recall	[0.03,0.73]	
							Precision	[0.79,1.00]	
							nCV_10-fold_	[0.84,0.86]	
						DT	Accuracy	[0.67,0.87]	
							AUC-ROC	[0.60,0.86]	
							Recall	[0.44,0.82]	
							Precision	[0.37,0.83]	
							nCV_10-fold_	[0.86,0.88]	
						Extra Trees	Accuracy	[0.82,0.86]	
							AUC-ROC	[0.88,0.94]	
							Recall	[0.58,0.77]	
							Precision	[0.67,0.82]	
							nCV_10-fold_	[0.86,0.88]	
						RF	Accuracy	[0.85.0.89]	
							AUC-ROC	[0.91,0.95]	
							Recall	[0.48,0.81]	
							Precision	[0.84,0.86]	
							nCV_10-fold_	[0.88,0.89]	
						CatBoost	Accuracy	[0.88,0.91]	
							AUC-ROC	[0.91,0.96]	
							Recall	[0.72,0.86]	
							Precision	[0.78,0.88]	
							nCV_10-fold_	[0.90,0.91]	
						GB	Accuracy	[0.88,0.89]	
							AUC-ROC	[0.90,0.95]	
							Recall	[0.66,0.84]	
							Precision	[0.81,0.85]	
							nCV_10-fold_	[0.89,0.90]	
						LightGBM	Accuracy	[0.82,0.90]	
							AUC-ROC	[0.88,0.96]	
							Recall	[0.68,0.85]	
							Precision	[0.63,0.87]	
							nCV_10-fold_	[0.88,0.90]	
						XGBoost	Accuracy	[0.85,0.90]	
							AUC-ROC	[0.88,0.96]	
							Recall	[0.61,0.85]	
							Precision	[0.72,0.87]	
							nCV_10-fold_	[0.89,0.90]	
Liao et al. (2024) [19]	Protein corona composition = f (NP properties, isolation, and formation of protein corona)	Metallic, liposomes, carbonaceous & others	Size_DLS_, Size_TEM_, zeta potential, PDI, NP shape, dispersion medium, dispersion medium pH	60 proteins	Not mentioned	RF	R^2^	[0.62,0.68]	tenfold cross-validation
							RMSE	[0.90,1.01]	

[a–b]: reported performance ranges from a to b in the paper

[a,b]: reported multiple performance ranges between a and b

~ [a,b]: approximately performance value inferred from figures has the values of a and b

## Featurization of Protein-Nanoparticle Interaction

The adsorption of proteins onto nanoparticles represents a multifactorial process governed by numerous physicochemical principles, and elucidating these interactions is critical for the rational design of nanoparticle-based drug delivery systems, diagnostics, and therapeutics, particularly for proteins, peptides, and vaccines. Analysis of the studies included in this review (Table I) indicates that protein adsorption is influenced by nanoparticle surface properties, protein characteristics, and environmental factors. Smaller nanoparticles have a higher surface area-to-volume ratio, offering more binding sites and affecting protein orientation, which can alter protein function. Nanoparticle shape also plays a role by presenting distinct surface facets and curvatures that may induce conformational changes impacting corona stability [19, 22–24, 81]. Surface charge, often measured as zeta potential, governs electrostatic interactions with proteins. Additionally, factors like ionic strength, chemical composition, and surface functionalization modulate adsorption strength and selectivity [24–28]. Similarly, protein characteristics such as size, surface charge (determined by isoelectric point), hydrophobicity, stability, flexibility, and surface accessibility influence adsorption strength and orientation [29, 90]. Environmental variables, including pH, ionic strength, temperature, protein concentration, and solvent composition, dynamically affect adsorption kinetics, protein conformations, and may lead to multilayer adsorption or aggregation [25, 27, 29, 30, 90].

Table II summarizes these NP, protein, and environmental features across the reviewed studies, providing a comprehensive reference for input variables in predictive modeling. Understanding these factors is essential for designing nanocarriers with controlled protein adsorption, which is critical for optimizing targeted delivery, reducing immunogenicity, and enhancing therapeutic efficacy. This framework establishes a foundation for machine learning-driven strategies to guide the rational design of nanocarriers in biologically relevant contexts, integrating dynamic environmental modeling with computational approaches to simulate real-world corona behavior and support the development of responsive, personalized nanomedicine platforms.

**Table 2:** Key Variables Influencing Protein-NP Interaction

Variable		Influence on Protein Adsorption
Nanoparticle Properties	Size	Surface area-to-volume ratio affects the number of binding sites and protein orientation; Smaller NPs lead to higher adsorption
	Shape	Curvature and surface facets influence protein binding and conformational changes
	Surface charge	Determines electrostatic interactions with proteins; positive zeta potential attracts negatively charged proteins
	Chemical composition or functional groups	Hydrophobicity and ligand chemistry modulate selective adsorption
	Surface modification	Stabilizes NPs and mediates specific protein interactions
Protein Properties	Size or molecular weight	Larger proteins offer more contact points for adsorption
	Surface charge or pI	Governs electrostatic interactions with NP surfaces
	Hydrophobicity	Drives adsorption to complementary NP surfaces
	Stability	Affects conformational adaptation upon binding
	Surface accessibility	Active binding sites increase adsorption strength
Environmental Factors	pH	Modulates protein charge and NP–protein interactions
	Ionic strength	Alters the electrostatic screening
	Temperature	Affects protein conformation and adsorption dynamics
	Protein concentration	Determines adsorption saturation and multilayer formation
	Solvent composition	Influences aggregation and adsorption layers

## Machine Learning Models for Protein-Nanoparticle Interactions

Over time, although traditional computational modeling approaches have provided valuable insights into protein-nanoparticle interactions, they have faced notable limitations, leading to a series of technological advancements. Although Langmuir and Freundlich isotherm models provided foundational insights into adsorption phenomena, they rely on simplistic assumptions of uniform surface properties and do not account for complex interactions or multilayer adsorption [91–93]. As computational power increased, molecular dynamics (MD) simulations emerged, offering detailed atomistic insights into protein-nanoparticle interactions. However, MD simulations are computationally intensive and heavily reliant on accurate force fields [93]. To address these limitations, coarse-grained models were developed, sacrificing atomic-level detail for computational efficiency [94]. Quantum mechanical calculations, particularly Density Functional Theory (DFT), brought unprecedented accuracy but were constrained by computational resources, limiting their application to smaller systems [95]. Machine learning (ML) algorithms then entered the scene, leveraging large datasets to predict interactions with high accuracy, albeit at the cost of interpretability [96, 97]. Monte Carlo simulations provided a complementary approach, exploring equilibrium properties but often overlooking dynamic behaviors [97, 98]. In addition to these, hydrophobic models were developed to capture the influence of hydrophobic interactions on protein-nanoparticle binding [98]. The BET (Brunauer–Emmett–Teller) models, widely used in surface chemistry, were also adapted to predict protein adsorption on nanoparticle surfaces [99]. While these techniques have advanced our understanding, they are not without drawbacks. QSAR models heavily depend on data quality and may lack generalization to new nanoparticle types. Moreover, the "black box" nature of ML models obscures underlying mechanisms, while coarse-grained models oversimplify interactions [100]. Overcoming these limitations will drive further innovation, leading to more robust and versatile methods for predicting protein-nanoparticle interactions.

In recent years, ML techniques have emerged as powerful tools for overcoming these limitations and revolutionizing the understanding and prediction of protein-nanoparticle interactions by harnessing vast datasets. Each study in Table I employs unique combinations of ML and DL architectures tailored to a specific research focus. For instance, linear and ε-SVR QSAR models predicted NP-cell association, employing MSE as the loss function, with protein corona fingerprints and physicochemical properties such as hydrodynamic size, zeta potential, and LSPR index as the input features [71]. As listed in Table I, similar input features and loss functions were later utilized by a subsequent study to leverage MLR and ANN in correlating liposome features with their biological response [22]. In the same year, another group utilized various architectures, including k-NN, SVM, RF, and PPR, to predict protein corona formation on gold nanoparticles based on LC–MS/MS spectral counts, employing MSE as the loss function [72]. MSE is one of the most widely used loss functions for regression tasks. It is mathematically defined as: $\frac{1}{n}\sum_{i=1}^{n}{({y}_{i}-{\widehat{y}}_{i})}^{2},$ where ${y}_{i}$ and ${\widehat{y}}_{i}$ represent the true and predicted values, respectively, and *n* is the number of samples. By calculating the average squared difference between them, MSE penalizes larger errors more heavily, which helps ensure precise predictions. However, this sensitivity to large deviations also makes it vulnerable to outliers. Its continuous and differentiable nature allows for efficient gradient-based optimization and stable convergence during model training.

In 2017, as mentioned in Table I, one study applied an ANN to quantify protein concentrations adsorbed on gold NPs using data of free and conjugated proteins, achieving a sensitivity of 92.5% and specificity of 100% [62]. Another study implemented WA, PLS, and RF architectures with structural descriptors and physicochemical and biological properties to predict NP toxicity, using MSE as the loss function [63]. Random Forest, the most frequently used model, was also applied to predict protein corona populations from LC–MS/MS protein data, utilizing cross-entropy loss for classification, resulting in an AUC of 0.83 and an F1-score of 0.81 [64]. Cross-entropy loss is typically used for classification problems. It measures how close the probability distribution of predicted class labels is to that of the true labels, defined as: $-\frac{1}{n}\sum_{i=1}^{n}[{y}_{i}\mathrm{log}\left({\widehat{y}}_{i}\right)+\left(1-{y}_{i}\right)\mathrm{log}(1-{\widehat{y}}_{i})]$. This function penalizes confident but incorrect predictions, encouraging the model to produce well-calibrated probability estimates. Cross-entropy generally leads to faster convergence and better interpretability when predicting class probabilities, which is particularly valuable for modeling binding events in protein coronas. Its main limitation is instability when predicted probabilities are extremely close to zero. A study in 2018 also employed cross-entropy as a loss function in the logistic regression model with MALDI-TOF mass spectrometry data as input to identify the threshold for peptide detection [76]. In 2019, ANN models applied cross-entropy for classification on DLS and MPT data, while MLR used MSE for regression tasks, consistent with other studies in Table I [66, 68]. Another example of Table I utilized both cross-entropy and MSE as loss functions, examining how sulfidation alters the protein corona formation on Ag and ZnO nanomaterials in human biological fluids, impacting exposure pathways, and utilizing the ubiquitous architecture RF model to predict protein corona compositions from protein features and NP characteristics [78]. In the same year, the influence of physicochemical characteristics on their toxicity was investigated using the RFDT model and MSE as the loss function [80]. Another recent study employed a supervised deep neural network (DNN) combined with mass spectrometry and meta-analysis to predict the functional composition of the protein corona and nanoparticle recognition by cells, providing additional insight into the *in vivo* fate of nanoparticles [100].

Random Forest (RF) is the most predominant model in protein corona prediction due to its ability to handle high-dimensional data and capture complex nonlinear relationships by averaging multiple decision trees, which reduces overfitting and improves predictive accuracy. RF also provides feature importance insights, helping to identify key variables that influence outcomes. Several studies, as highlighted in Table I, exploit these strengths to analyze protein corona compositions, using both Mean Squared Error (MSE) and Cross-Entropy as loss functions [81, 82]. A notable study used amino acid sequences from 131 antibodies, represented by 8,000 tripeptides, as input to a support vector machine (SVM) for binary classification, employing hinge loss [72]. Hinge loss is defined as: $\frac{1}{n}\sum_{i=1}^{n}\mathrm{max}(0, 1-{y}_{i}*{\widehat{y}}_{i})$, where the true labels ${y}_{i}$ are either + 1 or –1. This loss function focuses on maximizing the margin between classes, penalizing misclassified or boundary-violating samples, which improves generalization and creates a robust decision boundary. However, it is not suitable for multiclass or probabilistic models. An additional study generated 2646 images for 147 NPs for a CNN to predict physicochemical properties, with performance measured by R^2^ and RMSE (see Table I) [101]. Using L2 loss, which is MSE primarily used in regression tasks, various features of quantum dots and MexOy nanoparticles were analyzed using LightGBM to predict cytotoxicity further [85]. Research in 2022 used protein sequence data to predict protein adsorption on SWCNTs, employing multiple models, namely LGR and RF, with cross-entropy loss likely for classification tasks [86]. In 2023, multiple studies of Table I applied a DNN and various architectures to predict protein adsorption, measuring accuracy with R^2^, MSE, or cross-entropy loss function [87–89]. Finally, the research utilized various features, including size (DLS, TEM), zeta potential, PDI, NP shape, dispersion medium, and pH, to predict the composition of the protein corona on NPs. The study employed RF as the model, with the output being the prediction of protein corona composition, evaluated using R^2^ varying between 0.62 to 0.68 [19].

**Fig. 2:**
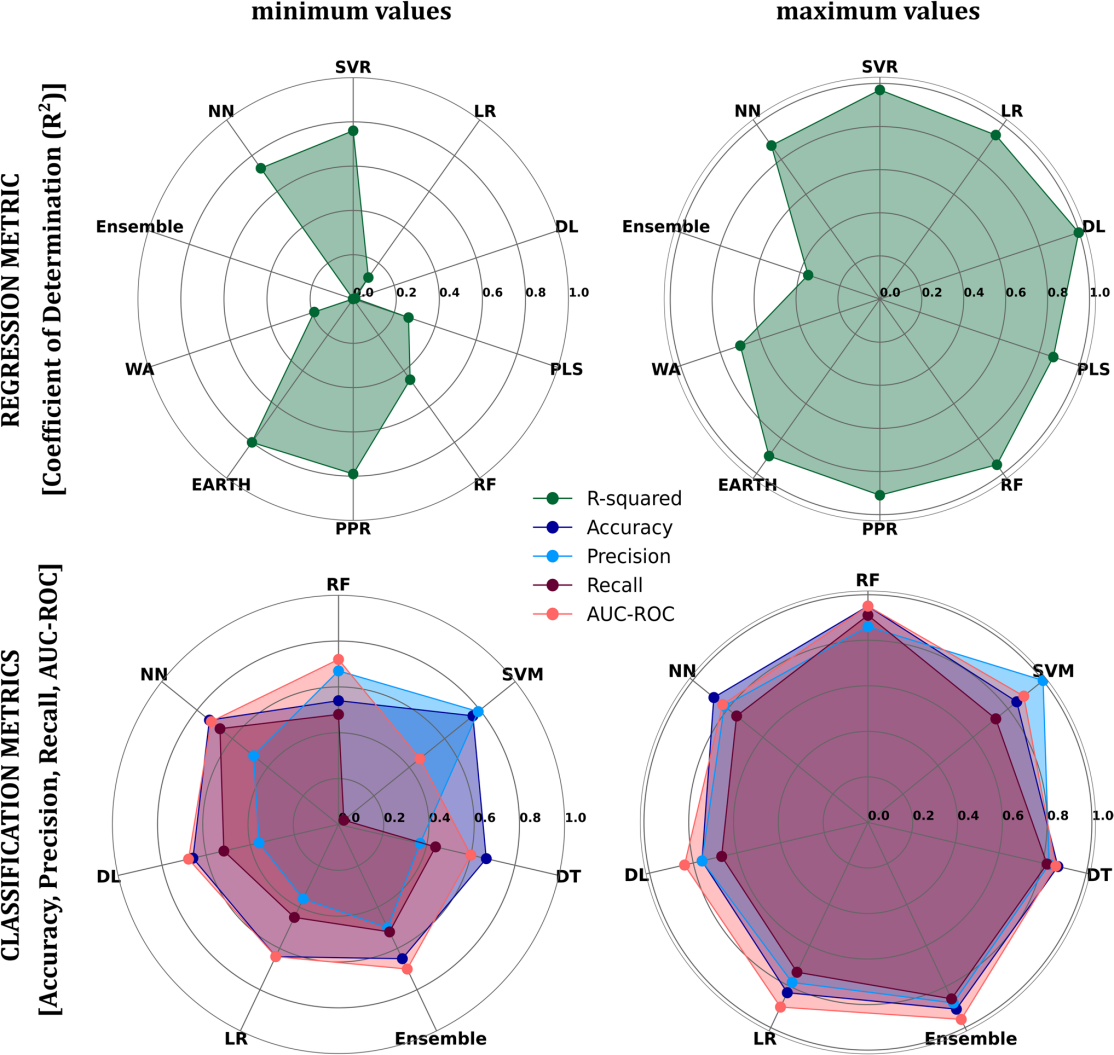
Represent the performance range (R^2^, Accuracy, Precision, Recall, and AUC-ROC) of each ML model.

To summarize, the studies reviewed and mentioned in Table I encompass a wide range of machine learning (ML) and deep learning (DL) approaches, along with various cross-validation techniques (Table I), for predicting the properties and behaviors of nanoparticles (NPs) and related biomolecules. Figure 2 illustrates the varying performances of the models, displaying the ranges of R^2^, accuracy, precision, recall, and AUC-ROC. The R^2^ of the DL models varied the most (0.01 to 0.99), while the EARTH (0.8–0.9) and PPR (0.79–0.91) demonstrated the least variability in R^2^. The accuracy of RF models varied from 0.54 to 0.95, and the ensemble models ranged from 0.65 to 0.91. Although RF had the narrowest precision range (0.67–0.86), the highest precision was obtained by SVM models. In contrast, SVM has the broadest recall range, and RF has the highest recall, 0.91. In addition, Fig. 2 shows the AUC-ROC of the models, where the RF and ensemble models' AUC-ROC ranged from 0.72 to 0.95 and 0.7 to 0.96, respectively.

## Databases

We also reviewed some of the major nanotechnology databases, which are crucial in advancing research and development in the field by providing comprehensive, structured, and accessible information on various nanomaterials and their properties. These databases facilitate data sharing, standardization, and the application of ML techniques to predict molecular properties and interactions.

The Public Virtual Nanostructure Simulation (PubVINAS) database (http://www.pubvinas.com/) contains detailed information on 705 unique nanomaterials across 11 material types. Each nanomaterial entry includes up to six physicochemical properties or bioactivities, resulting in over ten endpoints per entry. The database provides annotated nanostructures transformed into Protein Data Bank (PDB) files, which are available for download. Additionally, it features 2142 nanodescriptors for machine learning applications. The collection includes 414 gold nanoparticles (GNPs), 17 silver nanoparticles (AgNPs), 12 platinum nanoparticles (PtNPs), 12 palladium nanoparticles (PdNPs), 80 carbon nanotubes (CNTs), 48 buckminsterfullerenes (C60), 34 quantum dots (QDs), 32 metal oxides nanoparticles (MONPs), 21 DNA origami nanoparticles (DnaNPs), 11 dendrimers, and 24 cyclic peptide nanotubes (CPNTs). Properties of these nanomaterials include nanomaterial type, surface ligand number, size, logP, zeta potential, cell viability, reactive oxidative stress (ROS), and cellular uptake. The nanostructures can be viewed by the users online, or cheminformatics software such as VMD, RasMol, and MOE can be used to open the downloaded PDB files [101].

The cancer Nanotechnology Laboratory (caNanoLab) portal (https://cananolab.nci.nih.gov/) was initially developed to establish nanoparticle characterization standards and support data sharing among CCNEs and caBIG participants, with input from the NCL on the submission and retrieval of characterization data. The portal includes synthesis procedures, characterization protocols, and cancer nanotechnology-focused peer-reviewed publications. As of early 2022, the database comprises 1779 nanoparticle samples, 151 protocols, and 2253 publications, and the characterizations cover physicochemical, *in vitro*, and *in vivo* samples. The portal details various nanoparticle types, with metallic nanoparticles (26%) and polymeric nanoparticles (22%) being the most prevalent, followed by liposomes (10%). Other types include metalloids (such as silica), quantum dots, carbon-based nanomaterials (including nanotubes and fullerenes), dendrimers, emulsions, proteins, nucleic acids, biopolymers, and lipidic nanoparticles [102].

The Nanomaterial-Biological Interactions Knowledgebase (NBIK) (https://nbi.oregonstate.edu/) acts as a repository for annotated data on nanomaterial characterization, including purity, size, shape, charge, composition, functionalization, agglomeration state. It also covers synthesis methods and nanomaterial-biological interactions (beneficial, benign, or deleterious) defined at multiple levels of biological organization, from molecular to organismal [103].

The Nanowerk Nanomaterial Database (https://www.nanowerk.com/) is a comprehensive, free resource for the nanotechnology community. It provides access to information on approximately 4,500 nanomaterials, such as carbon nanotubes, nanoparticles, graphene, and quantum dots, from over 200 suppliers worldwide.

The Biomolecular Adsorption Database (BAD) (https://molecularsense.com/bad/) archives published protein adsorption data and is freely available online. It includes data on protein adsorption across nine types of surfaces, including polymers (49%), oxides (22.8%), modified silica (11.6%), silicon wafers (5.2%), phospholipids (4.2%), glass (3.9%), self-assembled monolayers (2%), gold (1.2%), and mica (0.3%). The database currently contains data on 25 representative proteins, with albumin, fibrinogen, lysozyme, immunoglobulin G, alpha-lactalbumin, and myoglobin being the most common [104].

Collectively, these databases provide a valuable foundation for understanding nanomaterial properties, interactions, and applications. As interest in nanoparticle-mediated delivery of protein therapeutics, subunit vaccines, and peptide-based immunotherapies continues to grow, structured and accessible data resources have become increasingly important. However, a major limitation lies in the lack of diversity, clinical relevance, and ML readiness of the available datasets. Most existing repositories remain heavily biased toward metallic and inorganic nanoparticles, while soft carriers such as liposomes and LNPs, which dominate current biologic delivery, are significantly underrepresented. Furthermore, datasets are often limited in size, suffer from inconsistent or incomplete annotations, and rarely capture corona formation profiles under physiologically relevant conditions or across different administration routes. These issues introduce bias and can undermine the assumptions made by ML models, such as correlations between features or uniform data distributions, potentially leading to overfitting or inaccurate predictions. Addressing these gaps is essential, as biased or unrepresentative data can significantly compromise model reliability and translational applicability. To overcome these challenges, future efforts must focus on expanding databases with biologically validated, standardized, and high-quality datasets on lipid-based and polymeric nanocarriers, enabling more accurate, diverse, and clinically relevant predictive modeling.

## Summary and Outlook

ML and QSAR models have revolutionized the understanding and prediction of complex interactions in nanomedicine, particularly protein-nanoparticle interactions. ML models, including supervised, unsupervised, semi-supervised, and reinforcement learning, offer powerful tools to handle large, complex datasets, providing accurate predictions and insights. Effective molecule representation plays a crucial role in these models, enabling the precise encoding of chemical and structural properties that facilitate the prediction of nanoparticle behaviors and interactions. Despite challenges, for instance, the need for high-quality data and model interpretability, advancements in computational techniques and interdisciplinary collaboration continue to drive innovation. Integrating machine learning with traditional modeling approaches enhances the design and optimization of nanoparticle-based systems for biomedical applications. Access to comprehensive nanotechnology databases further supports research and development, enabling the precise tailoring of nanomaterials for specific tasks. Addressing the curse of dimensionality in these large datasets is essential, as it can overwhelm traditional modeling techniques, hindering prediction accuracy. To mitigate this, surrogate models are being increasingly used to simplify high-dimensional data, enabling more efficient and accurate simulation of nanoparticle interactions. Moving forward, overcoming existing limitations and leveraging advanced modeling techniques will be crucial for further advancements in the field, ultimately improving the efficacy and safety of nanomedicine applications. The success of LNPs in mRNA vaccine delivery highlights the practical impact of well-characterized nanocarriers, underscoring the importance of understanding protein corona formation to optimize systemic exposure for biologic therapeutics.

The reviewed literature encompasses a broad spectrum of nanoparticles (NPs), including gold, silver, silica, maghemite, polystyrene, cadmium-containing quantum dots, zinc oxide, and cerium oxide, as well as engineered nanomaterials from resources such as the Nano-Tumor Database. Gold nanoparticles (Au-NPs) are among the most frequently studied, with variations including citrate-capped and surface-modified forms. In contrast, silver and ZnO NPs are often evaluated for corona formation, toxicity, and environmental interactions. Maghemite (SAMNs) have been explored for selective binding, polystyrene NPs (PS-COOH, PS-PEG) for microenvironment interactions, and cadmium quantum dots for their unique optical properties. This diversity reflects a wide interest in nanoparticle behavior across biological and environmental contexts. However, soft and clinically relevant nanocarriers, particularly liposomes and LNPs, remain underrepresented despite their increasing use in delivering biologics. These systems exhibit distinct interfacial properties and dynamic corona formation, underscoring the need for specialized modeling strategies. Critical nanoparticle features for predictive modeling include size (hydrodynamic, core), shape, surface charge (zeta potential), chemical modifications, surface area, LSPR, fluorescence, aspect ratio, and circularity. At the same time, protein attributes such as molecular weight, isoelectric point, amino acid composition, GRAVY score, and aliphatic index are equally important. Techniques such as DLS, TEM, LC–MS/MS, and ELISA provide essential data for these parameters; however, high-quality, standardized datasets remain scarce. Although major nanotechnology databases (PubVINAS, caNanoLab, NBIK, Nanowerk, BAD) compile extensive information, the limited availability of curated training data continues to hinder the development of robust machine learning (ML) models. Nevertheless, recent studies demonstrate the effectiveness of ML and deep learning in predicting protein corona formation, toxicity, and nanoparticle behavior, using loss functions such as Mean Squared Error (MSE) and cross-entropy. These findings highlight both the promise and current limitations of ML-guided approaches in advancing the design of safe and effective nanomedicine.

Several promising directions should be explored to enhance predictive modeling of nanoparticle-biological interactions, particularly for biologic and vaccine delivery. First, there is a critical need for high-quality, standardized experimental datasets focused on lipid-based and polymeric nanoparticles, especially those intended for protein, peptide, and RNA therapeutics. Expanding current databases to include corona profiles in clinically relevant biological fluids (e.g., plasma, lymph, mucus) and across different administration routes (e.g., intramuscular, mucosal) will improve model generalizability. Second, incorporating time-resolved corona evolution data and multi-omics information, such as transcriptomic or immunoproteomic readouts, will help predict downstream biological effects, including immunogenicity and therapeutic efficacy. Third, integrating mechanistic models (e.g., molecular dynamics or kinetic adsorption simulations) with data-driven machine learning frameworks yields hybrid models that are both accurate and interpretable. Future predictive modeling efforts should not focus solely on protein coronas; other biocorona, such as lipid coronas, are increasingly recognized for their roles in influencing nanoparticle fate, bio-distribution, and immunological responses. Incorporating these additional corona layers into machine learning pipelines will significantly enhance the accuracy and biological relevance of predicting nanoparticles' *in vivo* fate, especially for soft nanocarriers such as LNPs. Finally, future research should aim to develop unified, open-access platforms that integrate multiscale biocorona modeling with nanoparticle formulation optimization. Such tools enable the design of more comprehensive and mechanistically informed nanocarriers, thereby accelerating the development of personalized and targeted therapeutic systems. Together, these research directions will drive the next generation of effective, adaptive, and safe nanomedicine technologies.

## Conclusion

This review highlights the recent development of machine learning methods in predicting nanoparticle-protein interactions and the formation of protein corona. Our literature analysis points out that while ML models, especially Random Forest and Deep Learning, effectively infer complex correlations, their success heavily depends on the quality and quantity of training data. A key observation is the notable underrepresentation of soft, clinically relevant carriers, such as liposomes and LNPs, in publicly accessible databases, which hampers the development of reliable predictive models for protein corona formation. By highlighting the need for standardized, multi-dimensional datasets—including protein and lipid corona profiles, multi-omics data, and time-resolved dynamics—this work is hoped to shed light on advancing ML-guided nanomedicine design.

## Abbreviations

AdaBoost: Adaptive Boosting
AI: Artificial Intelligence
ANN: Artificial Neural Network
AUROC: Area under the Receiver Operating Characteristic Curve
BAC: Balance Accuracy
BAD: Biomolecular Adsorption Database
BET: Brunauer-Emmett-Teller
CatBoost: Categorical Boosting
CaNanoLab: Cancer Nanotechnology Laboratory
CD: Circular Dichroism
CNN: Convolutional Neural Network
CPANN: Counter Propagation Neural Network
CT: Computed Tomography
CV: Cross Validation
DFT: Density Functional Theory
DL: Deep Learning
DLS: Dynamic Light Scattering
DNN: Deep Neural Network
DT: Decision Tree
EARTH: Multivariate Adaptive Regression Splines
ELISA: Enzyme-Linked Immunosorbent Assay
ENM: Engineered Nanomaterial
EVS: Explained Variance Score
FTIR: Fourier-Transform Infrared
GB: Gradient Boosting
GRAVY: Grand Average of Hydropathy
GregNN: General regression Neural Network
GRU: Gated Recurrent Unit
HIC: Hydrophobic Interaction Chromatography
HP: Human Plasma
HSA: Human Serum Albumin
ICP-AES: Inductively Coupled Plasma-Atomic Emission Spectroscopy
k-NN: K-Nearest Neighbors
LightGBM: Light Gradient Boosting Machine
LC–MS/MS: Liquid chromatography-tandem mass spectrometry
LDA: Linear Discriminant Analysis
LGR: Logistic Regression
LR: Linear Regression
LSPR: Localized Surface Plasmon Resonance
LSTM: Long Short-Term Memory
MAE: Mean Absolute Error
MCC: Mathew Correlation Coefficient
MD: Molecular Dynamics
ML: Machine Learning
MLR: Multiple Linear Regression
MPT: Minute Particle Tracking
MRI: Magnetic Resonance Imaging
MS: Mass Spectrometry
MSE: Mean Squared Error
NBIK: Nanomaterial-Biological Interactions Knowledgebase
NP: Nanoparticle
PBPK: Physiologically Based Pharmacokinetic
PCA: Principal Component Analysis
PDB: Protein Data Bank
PET: Position Emission Tomography
PLS: Partial Least Squares
PPR: Projection Pursuit regression
PubVINAS: Public Virtual Nanostructure Simulation
QSAR: Quantitative Structure–Activity Relationship
RBFNN: Radial Basis Neural Network
RF: Random Forest
RFDT: Random Forest Decision Tree
RMSE: Root Mean Square Error
RNN: Recurrent Neural Network
ROS: Reactive Oxidative Stress
SAMN: Surface Active Maghemite NP
SCC: Somatic Cell Counts
SDS-PAGE: Sodium Dodecyl Sulphate–Polyacrylamide Gel Electrophoresis
SEM: Scanning Electron Microscopy
SGAC-SINSSalt-Gradient Affi: Nity-Capture Self-Interaction Nanoparticle Spectroscopy
sIgG: Sheep Immunoglobulin G
SMAC: Standup Monolayer Adsorption Chromatography
SSA: Specific Surface Area
SVM: Support Vector Machine
SVR: Support Vector Regression
SWCNT: Single-Walled Carbon Nanotube
TEM: Transmission Electron Microscopy
TES: Total Exposed Surface
TNN: Total Number of Nanoparticles
t-SNE: t-distributed Stochastic Neighbor Embedding
WA: Weighted Average
XGBoost: Extreme Gradient Boosting
